# Recent Advances in Occupational Exposure Assessment of Aerosols

**DOI:** 10.3390/ijerph17186820

**Published:** 2020-09-18

**Authors:** Martin Harper

**Affiliations:** 1 Zefon International, Inc., 5350 SW 1st Lane, Ocala, FL 34474, USA; mharper@zefon.com; 2 Department of Environmental Engineering Sciences, University of Florida, Black Hall, Gainesville, FL 32603, USA

**Keywords:** exposure assessment, aerosols, air sampling

## Abstract

Exposure science is underpinned by characterization (measurement) of exposures. In this article, six recent advances in exposure characterization by sampling and analysis are reviewed as tools in the occupational exposure assessment of aerosols. Three advances discussed in detail are (1) recognition and inclusion of sampler wall deposits; (2) development of a new sampling and analytical procedure for respirable crystalline silica that allows non-destructive field analysis at the end of the sampling period; and (3) development of a new sampler to collect the portion of sub-300 nm aerodynamic diameter particles that would deposit in human airways. Three additional developments are described briefly: (4) a size-selective aerosol sampler that allows the collection of multiple physiologically-relevant size fractions; (5) a miniaturized pump and versatile sampling head to meet multiple size-selective sampling criteria; and (6) a novel method of sampling bioaerosols including viruses while maintaining viability. These recent developments are placed in the context of the historical evolution in sampling and analytical developments from 1900 to the present day. While these are not the only advances in exposure characterization, or exposure assessment techniques, they provide an illustration of how technological advances are adding more tools to our toolkit. The review concludes with a number of recommended areas for future research, including expansion of real-time and end-of-shift on-site measurement, development of samplers that operate at higher flow-rates to ensure measurement at lowered limit values, and development of procedures that accurately distinguish aerosol and vapor phases of semi-volatile substances.

## 1. Introduction

Exposure science is underpinned by exposure assessment [1,2]. Exposure assessment includes modeling of exposures, but most importantly characterization (measurement) of exposures. In this article, six recent advances in sampling and analysis in the occupational exposure assessment of aerosols will be addressed. Three advances to be discussed in detail are (1) recognition and inclusion of sampler wall deposits; (2) development of a new sampling and analytical procedure for respirable crystalline silica that allows non-destructive field analysis at the end of the sampling period; and (3) development of a new sampler to collect the portion of sub-300 nm aerodynamic diameter particles that would deposit in human airways. Three additional developments will be described briefly: (4) a multi-fraction size-selective aerosol sampler; (5) a miniaturized pump with interchangeable sampling heads to meet different size-selective sampling criteria; and (6) a novel method of sampling bioaerosols including viruses while maintaining viability. In order to understand the rationale behind these recent advances, it is first necessary to recognize the historical context of prior advancements. Aerosols have been measured in the occupational environment since before 1900. Dust collection for investigation probably occurred even before the invention of the Aëroconiscope in 1870 [3]. Mining is one of the dustiest occupations and much research has been undertaken and many developments have arisen from consideration of aerosols in mining. The procedures in use at the beginning of the twentieth century are not the same as those used today. Changes have been driven by advances in our understanding of aerosol behavior and toxicity in air and in the human airways, and by technological innovations. One of the first procedures to be used in mining was to pull a measured volume of air through a tube containing granulated sugar [4]. The passage of humidified air caused the surface of the sugar to become sticky, trapping particles carried in the airstream. After sampling, the sugar could be dissolved and filtered off and the particles ignited to remove organic material. Then the particles could be segregated by size, if necessary, by sedimentation and then counted under a microscope in order to obtain a particle number concentration in particles per cubic centimeter or millions of particles per cubic foot. The sugar tube was used in mines prior to 1900 until its replacement by the Greenburg-Smith impinger in 1922 [5]. In this device, air is accelerated by passing through a nozzle immersed in water. The particles contained in the air have enough momentum to be carried to an impaction plate where their momentum is arrested and the particles are stopped, wetted and remain in the water for subsequent analysis. The original device operated at 1 cubic foot per minute (28.3 L min^−1^), but in 1934 it was miniaturized (the “midget impinger”) to operate at flow rates in the range of mL min^−1^ [6]. Impingers had the problem that they contained liquid, which was cumbersome in the field, but also it was known that very fine aerosol (original experiments used magnesium oxide fume and tobacco smoke) was not collected efficiently—such aerosol would attach to the surface of the air bubbles and be released through the bubbles bursting at the outlet. In 1944, the US Bureau of Mines investigated glass fiber filters [7], and in 1957, the US Public Health Service (the precursor of the National Institute for Occupational Safety and Health) published on the virtues of the polymeric membrane filters they had been using for several years [8], contained in brass holders similar to those being used by the US Atomic Energy Commission for airborne radionuclide sampling. Also, in the 1950s it was realized that it was the finer or “respirable” particles that were most toxic [9], so that size-selection curves were developed along with size-selective sampling devices (cyclones) for fine particle sampling. For several years, different respirable fractions were measured in different jurisdictions, which eventually were reconciled under ISO [10].

For larger particles than those considered for pneumoconiosis in mining, it was felt that all particles should be collected, and since the critical health issue is absorption and systemic toxicity, particle mass is considered more important than particle number (with the exception of mineral fibers). In 1956, a plastic cassette to house filters was developed for “clean-room” particle sampling, and, in 1960, it was featured in the first edition of the Air Sampling Instruments Handbook from the American Conference of Governmental Industrial Hygienists [11]. It was made by the Millipore corporation and known as the “Millipore Monitor”, but is now widely manufactured and is known more generally as the “closed-face cassette” (CFC), although it can also be used with a ring piece in place of the cap, where it is known as an “open-face cassette” (OFC). Both styles are manufactured to be used with filters of 25, 37, or 47 mm diameter. The cap on the CFC includes a small (4 mm) entry orifice, an advantage in preventing accidental or deliberate tampering with the filter, which was important since by that time pumps had been developed of size and weight suitable to be carried by a person for “personal sampling” [12]. It was believed that the cassette and filter, now known as a “sampler,” collected all particles in the air that would be relevant to inhaled dose onto the filter. The filter was the only part analyzed and considered to be an analysis of total particulate mass (TPM). However, only particles up to a certain size were collected efficiently [13]. Further research in the 1980s showed that larger particles up to 100 micrometers aerodynamic equivalent diameter (AED) could enter the nose and mouth while breathing [14]. Since mass is related to the cube of the diameter, even small numbers of large particles could have a profound impact on the inhaled mass dose. Note that most studies have considered solid particles; the behavior of liquid particles needs further study

The “inhalable” sampling convention developed out of this research [15] and an “inhalable” sampler was developed at the Institute of Occupational Medicine in Scotland, which became known as the IOM personal inhalable sampler [16]. During the development of this sampler it was clear that a substantial portion of the collected sample either did not reach the filter or bounced off the filter and was deposited on the internal surfaces [17]. The same issue affects other samplers, and its importance has now been studied over 30 years with clear conclusions bolstered by the research. That many occupational hygienists and the laboratories that serve them are either unaware of, or slow or even unwilling to accept the consequences is a puzzle.

## 2. Recognition and Inclusion of Sampler Wall Deposits

Samplers for aerosols typically consist of a filter or other collection substrate, for example an impaction plate or foam, supported in a container or holder. The entire device typically is considered an aerosol sampler. Part of the aerosol entering a sampler will deposit on the internal surfaces of the sampler prior to reaching the collection substrate. There are a number of mechanisms by which this can occur, including direct inertial impaction, gravitational settling, interception resulting from eddies during transport, electrostatic attraction and bounce from the filter [18]. All of these mechanisms can occur simultaneously, with the relative importance depending on factors such as particle size, shape, and density; inertial velocity; wind speed and orientation; etc. In addition, after sample collection, if the collection substrate is transported while mounted in the sampler, it is possible that particles originally deposited on the collection substrate may dislodge during transportation. Such particles can thereby contribute to deposits on the walls, as well as on the base of any cover plate or plug [19]. All particles found elsewhere than on or in the collection substrate are often loosely termed “wall deposits”. Deposits on internal surfaces are often invisible to the naked eye, even with transparent cassettes; they often comprise a large fraction of the aerosol that enters the sampler [20] (updated with additional information from the authors, personal communication, October 2007), [21,22,23,24] and can even exceed the quantity collected on the filter. Example data for CFCs are presented in Table 1. If the sample of interest entails the entire aspirated air particulate into the container or holder (sampler), it is necessary to account for these wall deposits, especially if it cannot be shown that they should be disregarded. Similar data for the IOM sampler are given in Table 2.

Most research exploring the extent of the wall loss phenomenon has considered inert particles [32,33], and metalliferous particulates [20,21,26,27,28,29,30,31]. However, the issue has also been studied for airborne organic materials, including bacterial endotoxin [34], wood [35], and pharmaceutical dusts [22]; another relevant study reported results from investigations in thermosetting plastics, wood, paper, and animal breeding [36]. Except in the case of very large wood dust particles, there is no evidence to suggest that wall deposited particles are sufficiently different from those found on the collection substrate to warrant their exclusion [37,38]. Wall deposits are not limited to aerosol samplers for larger airborne particles but may also be found in samplers for finer particles [39,40]. There is a justification for excluding wall deposits where the performance of an aerosol sampler tested to European Standard EN 13205 [41] shows appropriate compliance with the relevant ISO 7708 size-selective convention without their inclusion, but it should not be assumed. The sampling and analytical methods published in the *NIOSH Manual of Analytical Methods* (NMAM) represent state-of-the-art methods for assessing worker exposures to toxic chemicals. NIOSH considers that all particles entering the CFCs and the IOM sampler should be included as part of the sample whether they deposit on the filter or on the inside surfaces of the sampler. This is published policy [42] and is further stated in a section on the Manual web page. The US Occupational Safety and Health Administration (OSHA) has the same policy [43], which has specifically been addressed by their gravimetric method [44] since inception, and which is now explicitly written into new methods for metals sampling and analysis [45].

All aerosol particles entering an air sampler should be considered potential contributors to exposure, and this extends to gravimetric and chemical analyses. Appropriate sample preparation procedures are necessary to account for material on the internal surfaces. A number of techniques have been proposed, depending on the analytical finish: (1) sample extraction within the cassette; (2) rinsing of internal surfaces and adding the rinse to the filter preparation and analysis; (3) wiping the interior of the capsule and adding the wipe to the filter preparation and analysis; and (4) analysis of internal sampling capsules or cartridges (sampler inserts). Very few procedures have been developed and validated for carrying out sample extraction within the cassette. A procedure for in situ (that is, within-sampler) extraction in France [46] uses a 3-piece polystyrene cassette as the container for both sampling and extraction. Originally the filter used consisted of quartz fiber media, but the high background of some metals in these filters, together with the need for large quantities of hydrofluoric acid for complete dissolution of the filter, has led to replacement with a cellulose support pad and mixed cellulose ester (MCE) filter [47]. After sampling, the cassette is inverted and opened from the rear and the support pad removed, leaving the MCE filter in place. Acids (perchloric first, followed by nitric and hydrochloric, with hydrofluoric where necessary) are pipetted into the cassette, which is then sealed and placed in an ultrasonic bath for 10 min with the cassettes being upended after 5 min. Rinsing has not been shown to be a procedure with efficient recovery in most cases [43,47]. OSHA methods use wiping, for example with a clean “Smear Tab” (or 1 × 2 inch section of “Ghost Wipe”) that has been moistened with deionized water and placed in the same digestion beaker with any rinse of the interior and the sample filter [48]. The use of a single wipe without rinse has been validated by a NIOSH study [47]. However, wiping is cumbersome and potentially subject to operator variability. That leaves sampler inserts as the most practical and valid method for either gravimetric analyses or digestion with analysis for metals.

As noted above, OSHA uses a sampling method for gravimetric analysis, which is a CFC containing an internal capsule comprising a polyvinyl chloride (PVC) filter and aluminum foil cone. The cone has an inlet hole at its apex directly under the cassette inlet and the edge of the cone is tightly fixed to the filter. All particles are thus included on the filter or otherwise within the cone, which is pre- and post-weighed without removing the filter [44]. The same system is used with a cyclone pre-selector for respirable dust and respirable crystalline silica, in which case the filter is used to wipe the interior of the foil capsule before being digested for redeposition and analysis [49]. These capsules are expensive and a proposal for a cheaper all-PVC version (capsule and filter) was first suggested in 1992 for the collection of pharmaceutical dusts [50]. These sampler inserts are now available for both the 25 mm and 37 mm CFC and are known as Gravi-Serts™ (Zefon International, Inc., Ocala, FL, USA), and they can be used in NIOSH Method 0501 [51]. The Disposable Inhalable Sampler (DIS; Zefon International, Inc.) is a dimensional copy of the IOM sampler (patent pending) and similar PVC capsules and filters are available for the DIS. Since the geometric design of the DIS sampler is identical to that of the IOM sampler it has identical size-selective sampling performance characteristics. This was tested in bakeries with identical gravimetric results for side-by-side DIS and IOM samplers [52]. Cellulose acetate inserts with MCE filters have been extensively tested [30,53,54,55] and are intended for acid digestion and subsequent analysis for metals in NIOSH Method 7306 [56]. Commercially available products include Solu-Serts™ (Zefon International, Inc.) and Solu-CAPs™ (SKC Inc., Eighty Four, PA, USA). Again, a similar product is available for the DIS (Zefon International, Inc.). The DIS has other advantages over the IOM sampler—for example, it is inexpensive and intended for single use, thus avoiding the need for clean-up and the possibility of cross-contamination, an important concern for trace metal analysis. Background levels of the cellulosic DIS after microwave digestion in a combination of acids (nitric-hydrochloric-hydrofluoric) with hydrogen peroxide, by magnetic sector inductively coupled plasma-mass spectrometry are presented in Table 3.

NIOSH Method 0501 [51] was tested using blank internal capsules and with capsules spiked with 0.1–4 mg of National Institute of Standards and Technology (NIST) Standard Reference Material (SRM) 1648: Urban Particulate Matter, and with Arizona Road Dust (Air Cleaner Test Dust). The method reports a bias of 0.058 and an overall precision of 0.059, for an accuracy of ± 15.5%. Weight stability over 28 days was verified for both blanks and spiked capsules. Independent laboratory testing on blanks and field samples verified long-term weight stability and uncertainty estimates. The working range is given as 0.25 to 5 mg per sample, with an estimated Limit of Detection of 0.075 mg per sample and a precision of 0.031 at approximately 2 mg per sample. An inter-laboratory study of NIOSH Method 7306 [56] for 33 elements, gave recoveries better than 80% for all elements at both low and high spike levels, except for silver at the higher level. Accuracy of analysis, calculated from a combination of precision and bias, was 25% or less for all elements except silver. NIOSH Methods 0501 and 7306 should be used in place of older methods and the analyzing laboratory has a duty to inform their clients in this regard; ISO 17205 Section 7.1.2 states “The laboratory shall inform the customer when the method requested by the customer is inappropriate or out of date” [57]. Consideration of internal sampler wall deposits is included in related international voluntary consensus standards, published by ISO and ASTM International (formerly American Society for Testing and Materials), which describe the sampling and analysis of airborne metals and metalloids in occupational atmospheres.

Other large particle samplers have been shown to have internal deposits other than on the filter [25]. Samplers that collect both aerosols and vapor where, for example, the sampler consists of a filter cassette and sorbent tube in series may be similarly affected. Where cyclone-cassette assemblies are used for fine-particle, “respirable”, internal non-filter deposits are still found [39,40] but it is more difficult to account for them. Cassettes made of conductive materials dissipate the charges induced by charged fine particles, minimizing losses to the internal surfaces and should be used in place of non-conductive cassettes [42].

## 3. Bringing the Laboratory to the Field: On-Site Analysis of Respirable Crystalline Silica

Exposure to respirable fraction of crystalline silica (RCS) by inhalation can cause silicosis, lung cancer, other respiratory diseases, and kidney diseases [58]. Silica, especially quartz, is a common constituent of mined and quarried rocks and mineral deposits and materials used in construction including concrete, cement, bricks, aggregates, granite, slate and limestone. Exposure to RCS can occur during typical mining and construction. While the number of miners is relatively small, OSHA estimates about two million construction workers are exposed to RCS in over 600,000 US workplaces [59]. Studies of construction exposures have reported excessive exposures associated with certain tasks. For example, exposures ranging as high as 100 times the NIOSH recommended limit of 0.05 mg/m^3^ have been reported. The new OSHA comprehensive standard for silica in construction in effect from 23 September 2017, alongside a similar rule for general industry which took effect 23 June 2018, lowered the permissible exposure level (PEL) for RCS to 0.05 mg/m^3^ RCS and added a concentration level where exceedances would drive further enforcement actions (i.e., an “action level”) of 0.025 mg/m^3^ [59]. OSHA estimates that more than 840,000 construction workers are exposed to RCS levels that exceed the new PEL. OSHA’s Preliminary Economic Analysis and Initial Regulatory Flexibility Analysis expects a net benefit between $2.8 and 4.7 billion annually over the next 60 years by preventing between 579 and 796 annual fatalities from RCS exposure in all industries [59].

Demonstrating compliance with exposure limits for RCS requires the collection of respirable dust. Respirable dust is sampled from air using a cyclone or impactor to separate the respirable fraction from aerosol. Evaluation of RCS in the respirable dust has, until recently, required the use of off-site sophisticated laboratory analysis, but this can involve results being returned up to several weeks following the period sampled. Long lag times can lead to unacceptable conditions persisting during the interim without being recognized or addressed. While x-ray diffraction (XRD) is used extensively for the determination of silica in air samples, results from a proficiency test scheme for laboratories did not show any strong bias between XRD analysis and analysis by Fourier transform infra-red (FTIR) spectroscopy [60]. FTIR spectrometers today are manufactured sufficiently robust and small enough to be taken to the field allowing on-site analysis.

A methodology to quickly determine monitoring exposure results, even if the accuracy is outside of that required for compliance purposes, can be very useful. NIOSH has developed a field-ready methodology capable of an end-of-shift (EoS) measurement for RCS contained in airborne dusts in the mining sector [61], and this has been found also to provide results comparable to XRD [62]. RCS is collected on a direct-on-filter (DoF) sampler (EoS™ Silica Cassette, Zefon International, Inc.) attached to any one of several different cyclones to select the respirable dust fraction. Following sample collection, the cassette is removed from the cyclone and placed in a holder in any one of four different models of FTIR spectrometer, which have been evaluated for the purpose [63,64]. In a complex matrix of minerals, the estimation of quartz content may be subject to interference. Work is continuing to minimize these interferences by means of mine-specific correction factors [65]. However, since FTIR is a non-destructive analysis, samples can still be submitted to a laboratory for further analysis.

This method promises to be applicable to monitoring RCS in the construction sector. However, the construction samples contain not only silica but other components, which are typically different from those found in mines and quarries. The presence of such components also may interfere with the FTIR response from RCS in a manner which might require corrections to be applied to obtain a valid result. A recent pilot study evaluated possible interferences from different types of construction dusts—including drywall, plaster, cement, and brick—in laboratory-generated mixed dust samples [66]. Results from a set of prepared samples analyzed by portable FTIR showed that a) plaster and drywall dusts do not interfere substantially with the quartz measurement; b) cement does not interfere with the quartz measurement, but it does change the background absorbance of the filter; and c) in addition to having a substantial quartz content that has to be carefully evaluated in any study, brick dust may also contain an additional material, probably a silicate mineral, which interferes additionally with the quartz peak. In the range of interest from 20–110 µg per filter sample (bracketing the NIOSH REL/OSHA PEL for a 1 m^3^ air sample), 83% of the quartz contents predicted from the averaged calibration data agreed within 50% of the adjusted nominal loadings and 91% agreed within 100%. This result is encouraging given the high levels (500 or 1000 µg) of interfering dusts. An on-site reading of 100 µg is highly likely to be above the PEL, and a reading of 50 µg is highly likely to be above the action level so that appropriate action could be taken prior to receiving a definitive analysis from the laboratory. Samples loaded with smaller amounts of all four dusts in combination gave even better results, with all nine results within the range of interest falling within 25% of the adjusted nominal loadings. Both cement and brick could be correctable interferences once the identity of the interference is revealed, in the same way that correction is made for interfering mineral dusts in mines, and this is one of the further investigations suggested by this pilot study.

## 4. It’s All About the Size: How Should We Measure Exposure to Nanoparticles?

Exposure through inhalation of both incidental and engineered nanoparticles is a primary concern for worker health and safety, since nanoparticles are considered to have greater reactivity and thus toxicity compared to larger particles of similar composition [67]. Particles in this range can be engineered, for example, the metals and metal oxides and sulfides manufactured for commercial purposes, or incidental, including welding and other fumes. Although it was once thought that many sub-micrometer particles quickly agglomerate and attain particle sizes that are larger than 0.5 µm, there is growing evidence that a significant fraction of particles remains in singular or small number agglomerates in the nanoparticle (<100 nm) range. Around 300 nm there is a minimum in particle deposition in human airways, so that a particle of this size is far more likely to be breathed back out into the ambient atmosphere than to be deposited internally [68]. Below 300 nm the deposition efficiency begins to increase with decreasing particle size as a result of the importance of particle movement by diffusion resulting from Brownian motion, and interception. This deposition mechanism, unlike impaction or gravitational settling, occurs as much in the head airways as the lower respiratory tract.

Based initially on work on the collection efficiency of nylon screens [69], a lightweight (60 g), personal nanoparticle respiratory deposition sampler (NRD Sampler, Zefon International, Inc.) was developed to selectively collect particles smaller than 300 nm [70]. Most samplers are designed to sampling conventions that are based on penetration, but in this case the sampler was designed to collect nanoparticles with efficiency matching their deposition in the respiratory tract, in order to provide a physiologic relevance to sampler’s performance. A new sampling criterion was devised to provide the relevant deposition efficiency for the sampler [70]. The sampler operates at 2.5 Lpm and consists of a respirable cyclone fitted with an impactor and a diffusion stage. The cut-point diameter of the impactor is 300 nm with a sharpness σ = 1.53. The diffusion stage collects particles smaller than 300 nm according to the proposed convention. Impactor separation performance was not affected in experiments of loading at particle levels typically encountered in workplaces. The pressure drop of the NRD sampler is sufficiently low to permit its operation with conventional, belt-mounted sampling pumps. The initial design used nylon screens as the diffusion stage, and the limit of detection of common metals in welding fume using low-level spikes was determined to be 0.3 ug Ni, 0.4 ug Cr, and 0.9 ug Fe. Both laboratory [71] and field [72] trials in welding environments produced excellent results for short term samples, although it is possible that agglomerated particles, such as those which characterize welding fume, could with loading affect the porosity of the nylon screens, altering performance. Studies found that interception did become important as a collection mechanism as the collection of agglomerated nanoparticles progressed to higher loadings [73]. Performance of the nylon screens for agglomerated particles was found to be affected when the accumulated nanoparticle fraction loadings exceeded 1 mg. The change in performance also was accompanied by an increase in pressure drop across the screens to 14.3 kPa, and this could cause many commercial sampling pumps to fault. At the American Conference of Governmental Hygienists (ACGIH^®^) threshold limit value (TLV^®^) for welding fume of 5 mg/m^3^, a one-hour sample at 2.5 L min^−1^ collects 0.75 mg. The nanoparticle fraction of welding fume is typically less than half the total mass [70], so nylon screens are effective in sampling welding fume for one-hour or less. Nylon screens were also used for area samples of metal gouging and lancing side-by-side with inhalable, thoracic and respirable samplers [74]. Nanoparticles averaged around 170 nm in fume during gouging and around 30 nm during lancing. NRDS iron mass concentration was about 21% of inhalable for gouging and 28% for lancing. Unsurprisingly nanoparticles surface area dominated the total surface area.

The sampler now comes with a choice of two different diffusion stages. An alternative diffusion collection substrate, polyurethane foam, has characteristics more closely resembling human airways and may be preferable for collecting agglomerated materials, such as welding fume, in higher loading scenarios [70]. Polyurethane foam is easily digested in acids [75] and, unlike nylon, does not contain titanium allowing this sampler to be used to assess nanoparticle titanium dioxide [76]. Values for the median background of elements on small pieces of commercially available foam (*n* = 10) cleaned by a proprietary procedure, have been determined [77] after microwave digestion in a combination of acids (nitric-hydrochloric-hydrofluoric) with hydrogen peroxide, by magnetic sector inductively coupled plasma-mass spectrometry and are presented in Table 4. Results have been scaled to the size of the foam used in the NRD Sampler. Note that tin (approximately 10 micrograms per foam piece) is a cross-linking agent and is not removed by washing. The effective deposition to the foam was tested using sodium chloride aerosol, and up to 19 mg loading of metal fume was generated from welding rods using spark discharge. Field studies included testing against a device providing aerosol size distributions in three workplace situations [78]. Good correlations were found in the two workplaces (a heavy vehicle machining and assembly facility and a shooting range) where the aerosol was not dominated by large particles, but the correlation was not so good at an iron foundry where 95% of the particles were >1 µm aerodynamic equivalent diameter. Use of nanoparticle respiratory deposition samplers is described in the ASTM International (formerly American Society for Testing and Materials) Standard Practice [79].

## 5. Reducing Sample Numbers—Multi-Fraction Samplers

As noted, particles that penetrate to the gas-exchange region of the lungs are known as respirable. The diseases caused by inert respirable particles, including RCS, asbestos, coal dust, talc, bauxite, etc. cause, respectively, silicosis, asbestosis, “black lung”, talcosis and Shavers’ disease, known collectively as pneumoconiosis [80]. The exposures that cause these diseases traditionally have been assessed by measuring only those particles that penetrate to the gas-exchange region by means of a size-selector that only allows the fine particles to collect on the filter for analysis [81]. These size-selectors are designed to mimic size-selection in the lungs, and the most common technology is the miniature cyclone. On the other hand, particles of any size that can enter the mouth and nose that are soluble and can be absorbed, either in the lungs or in the gastrointestinal tract following expectoration and swallowing, can contribute to the dose of a systemic poison, for example, as with lead or cadmium. These particles are assessed by measurement of all particles that can enter the nose and mouth; a fraction termed “inhalable” [14]. Some metals are also thought to affect the lower reaches of the lung and so occupational exposure limit values for both inhalable and respirable fractions (e.g., for nickel compounds) are beginning to appear. There are many instances where knowledge of both inhalable and respirable particulates can be important and, traditionally, it has been necessary for the worker to wear two sets of sampling equipment (samplers and pumps) for the purpose of making the two determinations. Expensive multi-fraction samplers exist but are not widely used. A cheap alternative is the DIS with a foam insert as size-selector. With the correct type of foam, it is possible to match the ISO respirable convention. The filter collects the respirable faction, and anything between respirable and inhalable is caught in the foam, so that adding the analysis of the foam to that of the filter gives the inhalable fraction.

**Figure 1:**
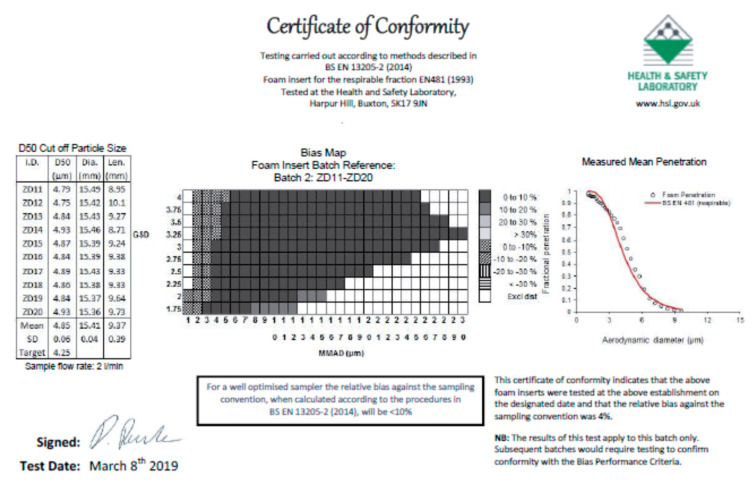
Certificate of conformity of disposable inhalable sampler (DIS) foam to EN13205 from the UK health and Safety Laboratory.

The size-selective performance of foams has been intensively studied and the concept of using foam has been thoroughly researched [82,83,84,85,86,87]. In a field study in several industries [86] the IOM dual-fraction sampler yielded similar results as personal cyclones with an explained variance (r^2^) of 0.8 and an association (β) of 0.93, not different from unity. The size-selective performance of the foam used in the DIS has been tested at the Health and Safety Executive in the UK. Over 96% of the aerosol size-distributions tested under EN13205 were separated by the DIS foam with less than 10% bias from ideal separation as shown in Figure 1.

A potential issue of using foam as a size-separator is the effect of progressive particle deposition within the foam on its separation characteristics. Penetration tests were carried out using IOM samplers with a respirable foam separator for 10 foams previously used to sample dust (Aloxite F800) in the laboratory at very high concentrations for short periods of time to produce loadings up to 50 mg, 20 foams previously used in sampling from a range of industries, selected to cover different particle types and dust loadings (on the foam) of up to 20 mg, and 3 blank foams [86]. A slight trend for the 50% cut-point to decrease with increasing loading was evident, but the effect was generally small, even with the exceptionally high loadings. The rapidly loaded laboratory samples were not more affected than the gradually loaded workplace samples. Loading effects were independent of particle type and small in comparison to the inherent variability of the foams. In a European study [87], two different foam plugs were tested with loading. The 10 mm diameter plugs with a mass load of 10 mg showed a decrease in D_50_ of some 7%, while 30 mm diameter plugs had a decrease of only 1%. Since the plugs in the DIS are 16 mm diameter, the expected reduction for a load of 10 mg is expected to be less than 5%. This study reported a limit of detection (LOD) of 0.015 mg based on 3 times standard deviation and a limit of quantitation (LOQ) of 0.050 mg based on 10 times standard deviation of foams similar to the DIS foam weighed in a glove box with controlled temperature (Peltier element with small fan) and saturated salt bath to control humidity. A different set of experiments in the same study but in a laboratory room with less control on the temperature and humidity gave an LOD of 0.069 mg and an LOQ of 0.230 mg.

Foam is easily soluble in an oxidizing acidic digestion [75]. The background and LOQ (based on 10 times the standard deviation of the blank) has been established through analysis by microwave digestion in a combination of acids (nitric-hydrochloric-hydrofluoric) with hydrogen peroxide, using magnetic sector inductively coupled plasma-mass spectrometry. Sodium (Na) and tin (Sn) are normal components of the foam and LOQs are 3.8 and 2.1 µg/foam, respectively. For other elements the results are presented in Table 5.

PVC filters are preferred for gravimetric analysis because they are more weight stable than other filters but do not digest entirely in acids, and this has led to the use of mixed cellulose-ester (MCE) filters in most published methods for metals. However, complete dissolution of the filter is not required so long as the dissolution of the sampled particles is efficient and the presence of undissolved filter does not compromise the sample entry to the instrument or interfere with the analysis. NIOSH has evaluated method 7304 [88] for processing PVC filters using microwave digestion and OSHA has a method [48] for processing PVC filters using hot-block digestion. While NIOSH showed that some elements such as antimony, silver, and tin do not form stable solutions in nitric acid when chloride from the PVC filters is present (and tin is also present in the background of PVC as a polymer cross-linking additive), these elements are not the most commonly evaluated in industrial hygiene investigations. While the OSHA method does not report any validation of the procedure, there is a back-up data report for the NIOSH Method documenting recovery [88]. In a test on PVC filters with PVC capsules, the Wisconsin State Hygiene Laboratory (Madison, WI, USA) was able to digest samples with a nitric-hydrochloric-hydrofluoric acid mixture plus hydrogen peroxide in a microwave oven. Although the background quantities of tin, sulfur and sodium are relatively high, all other elements analyzed were present below 1 µg, with many <1 ng. However, recovery studies are still to be performed. Using PVC filters allows for gravimetric analysis, and on-filter XRD or FTIR analysis for silica, prior to digestion for metals analysis. This leads to the possibility of a single, disposable (single use) sampler with determination of inhalable and respirable particulate, inhalable and respirable metals, and respirable crystalline silica.

## 6. Miniaturized Aerosol Samplers

Assessing personal exposure to air pollution is limited to a degree by the technology of samplers themselves. Wearable aerosol samplers are often noisy and burdensome, especially those designed to give real-time output of concentration, i.e., monitors. The ultrasonic personal aerosol (air) sampler (UPAS; Access Sensor Technologies, Fort Collins, CO, USA) has been developed to overcome many of these limitations [89]. The UPAS features a novel micropump with virtually silent operation. Onboard environmental sensors measure and record mass airflow (0.5–2.0 L min^−1^, accurate within 5%), temperature, pressure, relative humidity, light intensity, and acceleration. There is a mobile phone application available, which can be used to program and track the UPAS. Pump flow and pressure measurements, temperature and relative humidity, global positioning system coordinates, and semi-quantitative continuous particle mass concentrations based on filter differential pressure can be uploaded to a central server automatically whenever the mobile phone is connected to the internet, with sampled data automatically screened for quality control parameters. Filters and filter cartridges that are weighed pre- and post-sampling are barcoded to minimize sample collection errors and potentially track sources of contamination. Interchangeable cyclone inlets provide a close match to the EPA PM2.5 (particles below 2.5 μm in aerodynamic diameter) mass criterion (within 5%) for device flows at either 1.0 or 2.0 L min^−1^. Battery life varies from 23 to 45 h depending on sample flow rate and selected filter media. Laboratory tests of the UPAS prototype demonstrated excellent agreement with equivalent US federal reference method samplers for gravimetric analysis of PM2.5 across a broad range of concentrations [89]. Approximately 250,000 measurements of household and personal PM2.5 exposure using the UPAS were made in a multi-country cohort study [90], specifically to measure PM2.5 exposures for participants in rural communities in ten countries with high levels of indoor solid fuel use. Pilot study field evaluation of cooking area measurements indicated high correlation between the UPAS and reference Harvard Impactors (r = 0.91; 95% CI: 0.84, 0.95; slope = 0.95).

Additional inlets with collection efficiencies that match criteria for workplace respirable or thoracic mass sampling have also been developed and tested against polydisperse compressor oil aerosol [91]. The respirable mass inlet includes both an impaction stage and a cyclone, whereas the thoracic mass inlet utilizes a circular slot impactor. Both the respirable mass inlet and the thoracic mass inlet have been designed to be interchangeable with the PM2.5 inlet. Both inlets tested in the laboratory resulted in sample collection within ±5% of the respective criterion specifies for aerosols with reasonably broad size distributions. For dusts with mass median diameters smaller than about 6 µm the error in using the respirable inlet is under 5%. As is typical for respirable size-selectors, the error becomes larger with monodisperse dusts at larger particle sizes, but dusts with these characteristics are not commonly encountered. Bias of the thoracic inlet is generally within ±5% but increases to between 10 and 20% for dusts with median diameters between about 13 and 20 µm in diameter when the geometric standard deviation is under 2.0. Further validation work should include laboratory calibration with solid particles and characterization of field performance.

## 7. Bioaerosols and Viruses

Although the presence of viable microorganisms in air has been recognized for centuries [92], the development of quantitative sampling methods for bioaerosols only really took off in the 1990s when it was recognized that bacteria and fungi could play a role in building-related sickness [93]. The mix of species growing in damp buildings is typically different from the mix of outdoor species, so the development of sampling techniques focused primarily on the identification of bacterial and fungal species to detect problematic situations. In the absence of limit values, lesser attention was paid to the numbers, but viability was considered important in the assessment of pathogenic species, particularly spurred by the deliberate spread of anthrax bacteria in the USA. Unfortunately, many species of bacteria are fragile under the stress of normal impaction or filtration sampling [94] and specific techniques have been developed for the maximal preservation of viability [95]. Recent outbreaks of viral disease (SARS, H1N1, H1N5, MERS, SARS-Cov-2) have spurred investigations of airborne viruses.

The dynamics and significance of aerosol transmission of respiratory viruses are still controversial. One possible reason for this may be that collected viruses are inactivated by the collection method leading to inaccurate infection risk analyses. The viable virus aerosol sampler (BioSpot-VIVAS™ sampler; Aerosol Devices, Inc., Fort Collins, CO, USA) is a novel device designed to sample bioaerosols while preserving their viability to the greatest extent possible [96]. The VIVAS operates via a water vapor condensation process to enlarge aerosolized virus particles to facilitate their capture. It consists of eight parallel, wet-walled tubes termed growth tubes. During operation, the first half region of each tube is cooled to 6 °C, and the second half region is warmed to 45 °C. In the heated region, water vapor diffuses from the wet walls into the air stream faster than the air heats creating a supersaturation condition. Particles as small as 5 nm that can be wetted serve as condensation sites for the excess vapor from the supersaturated air and thus droplets are formed between approximately 10 µm and up to 2 mm in diameter. The flow exiting the tubes is directed by nozzles to a small volume of collection material in which the particles are collected, with a collection efficiency which was shown for inert particles from 8 nm to 10 µm to be greater than 95%. For viruses, the collection medium can be the same as that used typically for dilution in the generation of virus aerosols such as phosphate-buffered saline plus 0.5% (w/v) bovine serum albumin fraction V.

In an evaluation of the apparatus, fine aerosols (<500 nm) containing MS2 coliphage were generated from a Collison nebulizer, conditioned by a dilution dryer and collected by a VIVAS and a sampler commonly recommended for bioaerosol viability preservation, the BioSampler (SKC, Inc. Eighty Four, PA, USA). The VIVAS collected >93% of the inlet virus particles compared to about 10% for the BioSampler [97]. Viable counts of the VIVAS-collected viruses were also one order of magnitude higher than those of the BioSampler (*p* = 0.003), so that overall efficiency of the VIVAS exceeded that of the BioSampler for the viable collection of MS2 viruses by a factor of 10–100. Another set of experiments was performed using a more representative wild-type influenza, a H1N1 pandemic 2009 strain [98]. On average, the lower limit collection efficiency of the VIVAS was 74 ± 12% compared to 5.6 ± 3.0% for the BioSampler. The higher recovery from the VIVAS is attributed to the higher physical collection efficiency, slower rehydration of the infectious virus, and the gentle (non-destructive) impaction onto the collection medium.

A further pilot study was performed to determine whether infectious (viable) respiratory viruses in aerosols could be collected from air in a real-world environment and to determine the efficiency of viable collection compared to the BioSampler. A variety of viable human respiratory viruses, including influenza A H1N1 and H3N2 viruses and influenza B viruses, were collected by the VIVAS located at least 2 m from seated patients, during a late-onset 2016 influenza virus outbreak [99]. The BioSampler did collect virus aerosols, butt was considered less successful. These results using the VIVAS indicate that respiratory virus aerosols are more prevalent and potentially pose a greater inhalation biohazard than previously thought. The VIVAS thus appears to be a useful apparatus for microbiology air quality tests related to the detection of viable airborne viruses. In a very recent (not yet peer-reviewed) study with the VIVAS in a hospital, viable virus was isolated from air samples collected 2 to 4.8 m away from patients showing that patients with respiratory manifestations of COVID-19 produce aerosols that contain viable SARS-CoV-2, and these aerosols may serve as a source of transmission of the virus [100].

## 8. Conclusions

Anyone wishing to assess exposures to aerosols needs to understand that the science and technology exists in a continuum of research and response resulting in a ten- to twenty-year cycle of improving methodology; for example, from sugar tube to impinger to midget impinger to glass fiber filter to polymer membrane filter to closed-face cassette housing to “inhalable” samplers has taken about 100 years. Even more rapid recent advances have been made possible by new technologies. A selection of novel technologies described in more detail here includes procedures to include wall deposits, on-site analysis of silica, nanoparticles deposited in the airways, multi-fraction samplers, miniaturization of samplers and preservation of viability of microorganisms, especially viruses. This is only a sub-set of current developments, being those with which the author has had recent personal experience. It is expected that many of these and other research themes will continue in the future. Perhaps the most important theme is the development of samplers operating at higher flow rates [100], necessitated by lowered limit values and made possible by advances in pump and battery technology. It is likely that investigations will continue into real-time and end-of-shift on-site analyses, for example for silica [101] and metals [25]. Another situation for research is the characterization of semi-volatile aerosols. The equilibrium that exists between aerosol and vapor for semi-volatile substances has been known for a long time and there have been efforts to design samplers appropriate to the situation. However, recognition of the numbers of chemicals and situations where semi-volatile substances can occur has grown and resulted in further research into sampling methods [102]. The difficulty of obtaining an accurate separation of aerosol and vapor phases is profound, but the rewards would be a better understanding of physiological consequence of exposure and potential measures for controlling exposure, so, hopefully there will be continued research in this area. It is important to comprehend this continuum of research because it is not appropriate to ignore scientific advances and nor is it ethical to refuse to act upon them. Those who are reluctant to accept change because they mistakenly believe “things have always been done this way” need to recognize that “this is not your parent’s sampler” and likely, neither will it be your children’s.

## Figures and Tables

**Table 1:** Summary of findings of internal wall deposits as a percentage of total catch (filter plus walls) in field samples using the 37-mm CFC sampler.

Work Environment/Activity	*n*	Agent	Median Wall Deposit	Maximum Wall Deposit
Copper smelter [25]	18	Cu	21%	55%
Lead ore mill [26]	9	Pb	19%	35%
Solder manufacture [27]	30	Pb	29%	74%
Battery production [20]	16	Pb	28%	66%
Battery recycling [26]	54	Pb	29%	54%
Welding [28]	10	Cr(VI)	5%	55%
Plating [28]	12	Cr(VI)	12%	17%
Paint spray [28]	29	Cr(VI)	7%	12%
Zn foundry [20]	9	Zn	53%	62%
Zn plating [20]	18	Zn	27%	91%
Cast iron foundry [20]	18	Fe	22%	46%
Grey iron foundry [20]	18	Fe	24%	77%
Bronze foundry [29]	6	Cu, Pb, Zn	19%, 13%, 15%	45%, 17%, 21%
Cuproberyllium alloying [20]	4	Cu, Be	31%, 12%	40%, 39%
Solder manufacturer [30]	50	Pb	45%	77%
Solder manufacturer [30]	47	Sn	56%	93%

**Table 2:** Summary of findings of internal wall deposits as a percentage of total catch (filter plus walls) in field samples using the IOM sampler.

Work environment/Activity	*n*	Agent	Median Wall Deposit	Maximum Wall Deposit
Lead ore mill [26]	8	Pb	19%	30%
Copper smelter [25]	17	Cu	16%	38%
Copper refinery [19]	48	Cu	18%	36%
Battery production [31]	11	Pb	8%	33%
Welding [31]	18	Al	3%	13%
Cast iron foundry [20]	18	Fe	8%	69%
Grey iron foundry [20]	18	Fe	5%	16%
Bronze foundry [29]	6	Cu, Pb, Sn, Zn	0%, 0%, 0%, 3%	10%, 3%, 23%, 6%

**Table 3:** Blank levels of the cellulosic capsule of the DIS sampler (median of ten units, data from Wisconsin State Hygiene Laboratory, Madison, WI, USA).

Metal	Blank Levels [µg/Capsule]	Metal	Blank Levels [µg/Capsule]	Metal	Blank Levels [µg/Capsule]
Fe	0.41	Zn	0.024	Ce	0.0021
Al	0.36	Sn	0.018	Pb	0.0014
K	0.35	B	0.018	La	0.0012
Cr	0.34	Ni	0.012	As	0.0012
Ba	0.061	Cu	0.011	Ag	0.0011
Ti	0.058	Y	0.0071	V	0.00073
Mn	0.051	Hf	0.0028	Nb	0.00055
Se	0.030	Mo	0.0021	Co	0.00048

**Table 4:** Blank levels of elements in nanoparticle respiratory deposition (NRD) sampler foam (mean of ten foam pieces scaled to the size of that used in the sampler, data from Wisconsin State Hygiene Laboratory, Madison, WI, USA).

Metal	Blank Levels [µg/foam]	Metal	Blank Levels [µg/foam]	Metal	Blank Levels [µg/foam]
Al	1.9	B	0.139	Pb	0.018
Mg	1.8	W	0.057	Mo	0.016
Fe	1.4	Mn	0.056	Sr	0.014
Se	0.98	Ba	0.051	As	0.009
Ti	0.34	Ni	0.033	Co	0.006
Zn	0.3	Cr	0.028	Pt	0.004
Cu	0.15	V	0.019	Ce	0.003

**Table 5:** Limits of Quantitation (blank corrected) of elements in DIS sampler foam (mean of ten foam pieces, data from Wisconsin State Hygiene Laboratory, Madison, WI, USA).

Metal	LOQ [µg/Foam]	Metal	LOQ [µg/Foam]	Metal	LOQ [µg/Foam]
Ca	4.3	Cr	0.057	Cu	0.019
Fe	2.5	B	0.056	W	0.014
Zn	1.1	P	0.049	Sb	0.0054
K	1.0	Mo	0.035	V	0.0036
Al	0.96	Co	0.033	As	0.0034
Mg	0.43	Ni	0.028	Pb	0.0033
Ti	0.28	Ba	0.023	Pt	0.0030
Se	0.064	Mn	0.023	Sr	0.0010

## References

[1] Pleil J.D., Blount B.C., Waidyanatha S., Harper M. (2012). Establishing exposure science as a distinct scientific discipline. J. Expo. Sci. Environ. Epi..

[2] Harper M., Pleil J.D., Blount B.C., Miller A., Weis C., Hoover M.D., Jahn S. (2015). Commentary on a more comprehensive vision and strategy for the discipline of exposure science. J. Expo. Sci. Environ. Epi..

[3] Maddox R.L. (1870). On the apparatus for collecting atmospheric particles. Mon. Microscop. J..

[4] Fieldner A.C., Katz S.H., Longfellow E.S. (1921). The Sugar Tube Method of Determining Rock Dust in Air.

[5] Greenburg L., Smith G.W. (1922). A New Instrument for Sampling Aerial Dusts.

[6] Schrenck H.H., Feicht F.L. (1939). Bureau of Mines Midget Impinger.

[7] Brown C.E. (1944). Filter-Paper Method for Obtaining Dust-Concentration Results Comparable to Impinger Results.

[8] Paulus H.J., Talvitie N.A., Fraser D.A., Keenan R.G. (1957). Use of membrane filters in air sampling. Am. Ind. Hyg. Assoc. Q..

[9] Davies C.N. (1952). Dust sampling and lung disease. Br. J. Ind. Med..

[10] ISO (1983). Air Quality–Particle Size Fraction Definitions for Health-Related Sampling.

[11] American Conference of Governmental Industrial Hygienists (ACGIH) (1960). Air Sampling Instruments.

[12] Sherwood J. (1997). Realization, development and first applications of the personal air sampler. Appl. Occup. Environ. Hyg..

[13] Buchan R.M., Soderholm S.C., Tillery M.I. (1986). Aerosol sampling efficiency of 37-mm filter cassettes. Am. Ind. Hyg. Assoc. J..

[14] Armbruster L., Breuer H., Walton W.H. (1982). Investigations into defining inhalable dust. Inhaled Particles V.

[15] Vincent J.H., Armbruster L. (1981). On the quantitative determination of the inhalability of airborne dust. Ann. Occup. Hyg..

[16] Mark D., Vincent J.H. (1986). A new personal sampler for airborne total dust in workplaces. Ann. Occup. Hyg..

[17] Mark D. (1990). The use of dust-collecting cassettes in dust samplers. Ann. Occup. Hyg..

[18] Baron P.A., Ashley K. (2016). Chapter AE-Factors affecting aerosol sampling. NIOSH Manual of Analytical Methods (NMAM).

[19] Lee E.G., Grimson P.J., Chisholm W.P., Kashon M.L., He X., L’Orange C., Volckens J. (2019). Performance evaluation of disposable inhalable aerosol sampler at a copper electrorefinery. J. Occup. Environ. Hyg..

[20] Demange M., Gendre J.C., Hervé-Bazin B., Carton B., Peltier A. (1990). Aerosol evaluation difficulties due to particle deposition on filter holder inner walls. Ann. Occup. Hyg..

[21] Demange M., Görner P., Elcabache J.-M., Wrobel R. (2002). Field comparison of 37-mm closed-face filter cassettes and IOM samplers. Appl. Occup. Environ. Hyg..

[22] Puskar M.A., Harkins J.M., Moomey J.D., Hecker L.H. (1991). Internal wall losses of pharmaceutical dusts during closed-face, 37-mm polystyrene cassette sampling. Am. Ind. Hyg. Assoc. J..

[23] Dobson L., Reichmann L., Popp D. (2005). Evaluation of quartz residue on cassette interiors of AIHA proficiency samples. J. ASTM Int..

[24] Harper M., Demange M. (2007). Concerning sampler wall losses in the chemical analysis of airborne metals. J. Occup. Environ. Hyg..

[25] Harper M., Pacolay B., Hintz P.J., Bartley D.L., Slaven J.E., Andrew M.E. (2007). Portable XRF analysis of occupational air filter samples from different workplaces using different samplers: Final results, summary and conclusions. J. Environ. Monit..

[26] Harper M., Pacolay B., Hintz P., Andrew M.E. (2006). A comparison of XRF and ICP-OES for lead on air filter samples from a lead ore concentrator mill and a lead-acid battery recycler. J. Environ. Monit..

[27] Harper M., Pacolay B. (2006). A comparison of X-ray fluorescence and wet chemical analysis for lead on air filters from different personal samplers used in a secondary lead smelter/solder manufacturer. J. Environ. Monit..

[28] Occupational Safety and Health Administration (OSHA) (1983). Method ID215 (version 2): Hexavalent chromium. OSHA Analytical Methods Manual.

[29] Harper M., Pacolay B., Andrew M.E. (2005). A comparison of X-ray fluorescence and wet chemical analysis for lead on air filters from different personal samplers used in a bronze foundry. J. Environ. Monit..

[30] Lee E.G., Chisholm W.P., Burns D., Nelson J., Kashon M., Harper M. (2014). Comparison of lead and tin concentrations in air at a solder manufacturer from the closed-face 37 mm cassette with and without a custom cellulose-acetate cassette insert. J. Occup. Environ. Hyg..

[31] Hetland S., Thomassen Y. Sampling Efficiencies of the American 25-mm Personal Sampler. Presented at the Airmon: Modern Principles of Workplace Air Monitoring.

[32] Aitken R.J., Donaldson R. (1996). Large Particle and Wall Deposition Effects in Inhalable Samplers.

[33] Witschger O., Grinshpun S.A., Fauvel S., Basso G. (2004). Performance of personal inhalable aerosol samplers in very slowly moving air when facing the aerosol source. Ann. Occup. Hyg..

[34] Duquenne P., Simon X., Demange V., Harper M., Wild (2015). P. Endotoxin deposits on the inner surfaces of closed-face cassettes during bioaerosol sampling: A field investigation at composting facilities. Ann. Occup. Hyg..

[35] Li S.N., Lundgren D.A., Rovell-Rixx D. (2000). Evaluation of six inhalable aerosol samplers. Am. Ind. Hyg. Assoc. J..

[36] Lidén G., Melin B., Lidblom A., Lindberg K., Norén J.-O. (2000). Personal sampling in parallel with open-face filter cassettes and IOM samplers for inhalable dust. Implications for occupational exposure limits. Appl. Occup. Environ. Hyg..

[37] Lee T., Chisholm W.P., Slaven J.E., Harper M. (2009). Size distributions of 0.5 to 20 µm aerodynamic diameter lead-containing particles from aerosol sampler walls and filters. Aerosol Sci. Technol..

[38] Chisholm W.P., Lee T., Slaven J.E., Nelson J., Harper M. (2011). Comparison of filter and wall deposits from samplers used to collect airborne lead-containing dusts at field sites. Aerosol Sci. Technol..

[39] Dobson L., Reichmann L., Popp D., Harper M., Lee T. (2013). Evaluation of Quartz Residue on Cassette Interiors of AIHA Proficiency Samples. Silica and Associated Respirable Mineral Particles.

[40] Soo J.-C., Monaghan K., Lee T., Kashon M., Harper M. (2016). Air sampling filtration media: Collection efficiency for respirable size-selective sampling. Aerosol Sci. Technol..

[41] Comité Européen de Normalization (CEN) (2014). EN 13205, Workplace exposure. Assessment of Sampler Performance for Measurement of Airborne Particle Concentrations (In Four Parts).

[42] Ashley K., Harper M. (2013). Closed face filter cassette (CFC) sampling—Guidance on procedures for inclusion of material adhering to internal sampler surfaces. J. Occup. Environ. Hyg..

[43] Hendricks W., Stones F., Lillquist D. (2009). On wiping the interior walls of 37-mm closed-face cassettes: An OSHA perspective. J. Occup. Environ. Hyg..

[44] Occupational Safety and Health Administration (OSHA) (1983). Method PV2121: Gravimetric determination; updated 2003. OSHA Analytical Methods Manual.

[45] Institut National de Recherche et de Sécurité pour la Prévention des Accidents du Travail et des Maladies Professionnelles (INRS) (2005). Métaux et métalloïdes, M-124. MétroPol.

[46] Demange M., Elcabache J.-M., Boulet A. (2003). Mise en solution à froid des membranes en ester de cellulose dans le cadre de l’analyse des aérosols. Can. J. Anal. Sci. Spectr..

[47] Ceballos D., King B., Beaucham C., Brueck S.E. (2015). Comparison of a wipe method with and without a rinse to recover wall losses in closed face 37-mm cassettes used for sampling lead dust particulates. J. Occup. Environ. Hyg..

[48] Occupational Safety and Health Administration (OSHA) (1983). Method ID 125G: Metal and metalloid particulates in workplace atmospheres (ICP analysis); updated 2002. OSHA Analytical Methods Manual.

[49] Occupational Safety and Health Administration (OSHA) (1983). Method ID 142: Crystalline silica (Quartz and cristobalite); version 4, 2016. OSHA Analytical Methods Manual.

[50] Puskar M.A., Fergon S.M., Harkins J.M., Hecker L.H. (1992). Gravimetric determination of airborne dust by using a filter cartridge inside a closed-face, 37-mm polystyrene cassette. Am. Ind. Hyg. Assoc. J..

[51] Ashley K., National Institute for Occupational Safety and Health (NIOSH) (2015). Method 0501, Particulates Not Otherwise Regulated, Total. NIOSH Manual of Analytical Methods (NMAM).

[52] Institut de Recherche Robert-Sauvé en Santé et en Sécurité du Travail du Québec (IRSST) (2019). INFO-LABO 2019-06: La Cassette Inhalable: Nouveau Média d’Échantillonnage pour les Poussières de Fraction Inhalable.

[53] Harper M., Ashley K. (2012). Preliminary studies on the use of acid-soluble cellulose acetate internal capsules for workplace metals sampling and analysis. J. Occup. Environ. Hyg..

[54] Harper M., Ashley K. (2013). Acid-soluble internal capsules for closed-face cassette elemental sampling and analysis of workplace air. J. Occup. Environ. Hyg..

[55] Andrews R.N., Feng H.A., Ashley K. (2016). Interlaboratory evaluation of cellulosic acid-soluble internal air sampling capsules for multi-element analysis. J. Occup. Environ. Hyg..

[56] Ashley K., National Institute for Occupational Safety and Health (NIOSH) (2015). Method 7306, Elements by Cellulosic Internal Capsule Sampler. NIOSH Manual of Analytical Methods (NMAM).

[57] ISO/IEC (2017). 17205—General Requirements for the Competence of Testing and Calibration Laboratories.

[58] Steenland K. (2005). One agent, many diseases: Exposure-response data and comparative risks of different outcomes following silica exposure. Am. J. Industr. Med..

[59] Occupational Safety and Health Administration (OSHA), Department of Labor (2016). Occupational Exposure to Respirable Crystalline Silica, Final Rule. Fed. Regist..

[60] Harper M., Sarkasian K., Andrew M. (2014). Assessment of respirable crystalline silica analysis using proficiency analytical testing results from 2003–2013. J. Occup. Environ. Hyg..

[61] Cauda E., Miller A., Drake P. (2016). Promoting early exposure monitoring for respirable crystalline silica: Taking the laboratory to the mine site. J. Occup. Environ. Hyg..

[62] Miller A.L., Drake P.L., Murphy N.C., Noll J.D., Volkwein J.C. (2012). Evaluating portable infrared spectrometers for measuring the silica content of coal dust. J. Environ. Monit..

[63] Hart J.F., Autenrieth D.A., Cauda E., Chubb L., Spear T.M., Wock S., Rosenthal S. (2018). A comparison of respirable crystalline silica concentration measurements using a direct-on-filter Fourier transform infrared (FT-IR) transmission method versus a traditional laboratory X-ray diffraction method. J. Occup. Environ. Hyg..

[64] Ashley E.L., Cauda E., Chubb L.G., Tuchman D.P., Rubinstein E.N. (2020). Performance comparison of four portable FTIR instruments for direct-on-filter measurement of respirable crystalline silica. Ann. Work Exp. Health.

[65] Cauda E., Chubb L., Reed R., Stepp R. (2018). Evaluating the use of a field-based silica monitoring approach with dust from copper mines. J. Occup. Environ. Hyg..

[66] Chien C.-H., Huang G., Lopez B.M., Morea A.F., Sing S.Y., Wu C.Y., Kashon M.L., Harper M. (2020). Application of end-of-shift respirable crystalline silica monitoring to construction. J. Occup. Environ. Hyg..

[67] Papp T., Schiffmann D., Weiss D., Castranova V., Vallyathan V., Rahman Q. (2008). Human health implications of nanomaterial exposure. Nanotoxicology.

[68] International Commission on Radiological Protection (ICRP) (1994). Human Respiratory Tract Model for Radiological Protection.

[69] Cena L.G., Ku B.K., Peters T.M. (2012). Particle collection efficiency for nylon mesh screens. Aerosol Sci. Technol..

[70] Cena L.G., Anthony T.R., Peters T.M. (2011). A personal nanoparticle respiratory deposition (NRD) sampler. Environ. Sci. Technol..

[71] Cena L.G., Keane M.J., Chisholm W.P., Stone S., Harper M., Chen B.T. (2014). A novel method for assessing respiratory deposition of welding fume nanoparticles. J. Occup. Environ. Hyg..

[72] Cena L.G., Chisholm W.P., Keane M.J., Chen B.T. (2015). A field study on the respiratory deposition of the nano-sized fraction of mild and stainless steel welding fume metals. J. Occup. Environ. Hyg..

[73] Mines L.W.D., Park J.H., Mudunkotuwa I.A., Anthony T.R., Grassian V.H., Peters T.M. (2016). Porous polyurethane foam for use as a particle collection substrate in a nanoparticle respiratory deposition sampler. Aerosol Sci. Technol..

[74] Keyter M., Van Der Merwe A., Franken A. (2019). Particle size and metal composition of gouging and lancing fumes. J. Occup. Environ. Hyg..

[75] Dillner A.M., Shafer M.M., Schauer J.J. (2007). A novel method using polyurethane foam (PUF) substrates to determine trace element concentrations in size-segregated atmospheric particulate matter on short time scales. Aerosol Sci. Technol..

[76] Mudunkotuwa I., Anthony T.R., Grassian V., Peters T.M. (2015). Accurate quantification of TiO_2_ nanoparticles collected on air filters using a microwave-assisted acid digestion method. J. Occup. Environ. Hyg..

[77] Harper M., Peters T. Sampling and Analysis of Sub-micron Metal Particulate for Respiratory Dose Estimation. Proceedings of the ASTM International Conference on Measurement of Trace Metals and Metalloids at Workplaces.

[78] Stebounova L.V., Gonzalex-Pech N.I., Park J.H., Anthony T.R., Grassian V.H., Peters T.M. (2018). Particle concentrations in occupational settings measured with a nanoparticle respiratory deposition (NRD) sampler. Ann. Work Expo. Health.

[79] American Society for Testing and Materials (ASTM International) (2019). D8208, Standard Practice for Collection of Non-Fibrous Nanoparticles Using a Nanoparticle Respiratory Deposition (NRD) Sampler.

[80] Morgan W.K.C., Seaton A. (1995). Occupational Lung Diseases.

[81] Lippmann M., Harris W.B. (1962). Size selective samplers for estimating the “respirable” dust concentrations. Health Phys..

[82] Aitken R.J., Vincent J.H., Mark D. (1993). Application of porous foams as size selectors for biologically relevant samplers. Appl. Occup. Environ. Hyg..

[83] Chung K.Y.K., Aitken R.J., Bradley D.R. (1997). Development and testing of a new sampler for welding fume. Ann. Occup. Hyg..

[84] Chen C.C., Lai C.Y., Shih T.S., Yeh W.Y. (1998). Development of respirable aerosol samplers using porous foams. Am. Ind. Hyg. Assoc. J..

[85] Breum N.O. (2000). The dust holding capacity of porous plastic foam used in particle size-selective sampling. J. Aerosol Sci..

[86] Kenny L., Chung K., Dilworth M., Hammond C., Wynn Jones J., Shreeve Z., Winton J. (2001). Applications of low-cost, dual-fraction dust samplers. Ann. Occup. Hyg..

[87] Aitken R.J., Görner P., Kenny L.C., Möhlmann C., Vu Duc T., Zambelli G. (2002). Project “Size Selective Personal Air Sampling Using Porous Plastic Foams” (PoFAS).

[88] Ashley K., National Institute for Occupational Safety and Health (NIOSH) (2014). Method 7304, Elements by ICP (Microwave digestion). NIOSH Manual of Analytical Methods (NMAM).

[89] Volckens J., Quinn C., Leith D., Mehaffy J., Henry C.S., Miller-Lionberg D. (2017). Development and evaluation of an ultrasonic personal aerosol sampler. Indoor Air.

[90] Arku R.E., Birch A., Shupler M., Yusuf S., Hystad P., Brauer M. (2018). Characterizing exposure to household air pollution within the prospective urban rural epidemiology (PURE) study. Environ. Int..

[91] Leith D., L’Orange C., Mehaffy J., Volckens J. (2020). Design and performance of UPAS inlets for respirable and thoracic mass sampling. J. Occup. Environ. Hyg..

[92] Carnelly T., Haldane J.S., Anderson A.M. (1887). The carbonic acid, organic matter and micro-organisms in air, more especially of dwellings and schools. Phil. Trans. R. Soc. Lond. B.

[93] Dowes J., Thorne P., Pearce N., Heedrick D. (2003). Bioaerosol health effects and exposure assessment: Progress and prospects. Ann. Occup. Hyg..

[94] Yao M., Mainelis G. (2007). Analysis of portable impactor performance for enumeration of viable aerosols. J. Occup. Environ. Hyg..

[95] Lin X., Reponen T., Willeke K., Wang Z., Grinshpun S.A., Trunov M. (2000). Survival of microorganisms during swirling aerosol collection. Aerosol Sci. Technol..

[96] Lednicky J., Pan M., Loeb J., Hsieh H., Eiguren-Fernandez A., Hering S., Fan Z.H., Wu C.-Y. (2016). Highly efficient collection of infectious pandemic influenza H1N1 virus (2009) through laminar-flow water based condensation. Aerosol Sci. Technol..

[97] Pan M., Eiguren-Fernandez A., Hsieh H., Ashfar-Mohajer N., Hering S., Lednicky J., Fan Z.H., Wu C.-Y. (2016). Efficient collection of viable virus aerosol through laminar-flow, water-based condensational particle growth. J. Appl. Microbiol..

[98] Pan M., Bonny T.S., Loeb J., Jiang X., Lednicky J.A., Eiguren-Fernandez A., Hering S., Fan Z.H., Wu C.-Y. (2017). Collection of viable aerosolized influenza virus and other respiratory viruses in a student health care center through water-based condensation growth. Msphere.

[99] Lednicky J.A., Lauzardo M., Fan Z.H., Jutla A., Tilly T.B., Gangwar M., Usmani M., Shankar S.N., Mohamed K., Eiguren-Fernandez A. (2020). Viable SARS-CoV-2 in the air of a hospital room with COVID-19 patients. Medrxiv.

[100] Lee T., Lee E.G., Kim S.W., Chisholm W.P., Kashon M., Harper M. (2012). Quartz measurement in coal dust with high flow rate samplers: Laboratory study. Ann. Occup. Hyg..

[101] Zheng L., Kulkarni P., Birch M.E., Ashley K., Wei S. (2018). Analysis of crystalline silica aerosol using portable Raman spectrometry: Feasibility of near real-time measurement. Anal. Chem..

[102] Breuer D., Dragan G.C., Friedrich C., Möhlmann C., Zimmermann R. (2015). Development and field testing of a miniaturized sampling system for simultaneous sampling of vapours and droplets. Environ. Sci. Process. Impacts.

